# Pathogenesis of Ischemic Stroke: Role of Epigenetic Mechanisms

**DOI:** 10.3390/genes11010089

**Published:** 2020-01-13

**Authors:** Rosita Stanzione, Maria Cotugno, Franca Bianchi, Simona Marchitti, Maurizio Forte, Massimo Volpe, Speranza Rubattu

**Affiliations:** 1 IRCCS Neuromed, Via Atinense, 18, 86077 Pozzilli IS, Italy; maria.cotugno@neuromed.it (M.C.); franca.bianchi@neuromed.it (F.B.); simona.marchitti@neuromed.it (S.M.); maurizio.forte@neuromed.it (M.F.); massimo.volpe@uniroma1.it (M.V.); rubattu.speranza@neuromed.it (S.R.); 2 Department of Clinical and Molecular Medicine, School of Medicine and Psychology, Sapienza University of Rome, 00189 Rome, Italy

**Keywords:** ischemic stroke, epigenetic, DNA methylation, histone modification, miRNA, lncRNA

## Abstract

Epigenetics is the branch of molecular biology that studies modifications able to change gene expression without altering the DNA sequence. Epigenetic modulations include DNA methylation, histone modifications, and noncoding RNAs. These gene modifications are heritable and modifiable and can be triggered by lifestyle and nutritional factors. In recent years, epigenetic changes have been associated with the pathogenesis of several diseases such as diabetes, obesity, renal pathology, and different types of cancer. They have also been related with the pathogenesis of cardiovascular diseases including ischemic stroke. Importantly, since epigenetic modifications are reversible processes they could assist with the development of new therapeutic approaches for the treatment of human diseases. In the present review article, we aim to collect the most recent evidence concerning the impact of epigenetic modifications on the pathogenesis of ischemic stroke in both animal models and humans.

## 1. Introduction

Ischemic stroke (IS) represents the second global cause of death, after ischemic heart disease, and the most common cause of disability worldwide [1,2,3]. IS occurs when the occlusion of a cerebral artery by an embolus or a thrombus produces a reduction of blood flow and oxygen to the brain [4]. This phenomenon, when persistent, contributes to irreversible brain damage due to neuronal cell death. The increase of both inflammation and oxidative stress is another common feature of IS, and can be interpreted either as a cause or as a consequence [5]. Stroke is a heterogeneous, multifactorial disease resulting from the interaction between numerous environmental and genetic risk factors [6,7]. Several case-control association studies revealed the contributory role of single nucleotide polymorphisms (SNPs) belonging to key hormonal and molecular systems including: the renin-angiotensin-aldosterone system (RAAS) [8], the natriuretic peptides family [9], the hemostasis and coagulation cascade [10,11], the lipoprotein metabolism [12], inflammation [13,14], homocysteine metabolism [15]. Our group has demonstrated that genes encoding proteins of the mitochondrial electron transport chain are key elements to guarantee correct mitochondrial function, cell viability and protection from stroke occurrence [16,17,18,19,20]. Genome-wide association studies confirmed the existence of several variants belonging to different genes associated with IS and their specific subtypes, such as paired-like homeodomain transcription factor 2 (*PITX2*) and Zinc Finger Homeobox 3 (*ZFHX3*) in the cardioembolic stroke [21], histone deacetylase 9 (*HDAC9*), and cyclin dependent kinase inhibitor (*CDKN*) in the large-vessel stroke subtype [22].

Although several studies have investigated the molecular and genetic mechanisms underlying the pathogenesis of stroke, additional mechanisms still remain to be elucidated. Recently, increased evidence supports the involvement of epigenetic alterations in the pathogenesis of cardiovascular diseases in both humans and animal models [23,24], and particularly in the susceptibility to stroke.

Epigenetic modifications alter gene expression without changing the DNA sequence. Some of them are stable and inherited by subsequent generations [25]. In several cases, the epigenetic changes are dynamic and responsive to environmental stimuli. Epigenetic mechanisms can regulate different physiological processes that occur in a living organism, including cellular proliferation and differentiation. A deeper knowledge of the epigenetic modifications related to stroke and to its risk factors could provide the basis for the development of innovative approaches in the prevention and treatment of this disabling disease.

**Figure 1:**
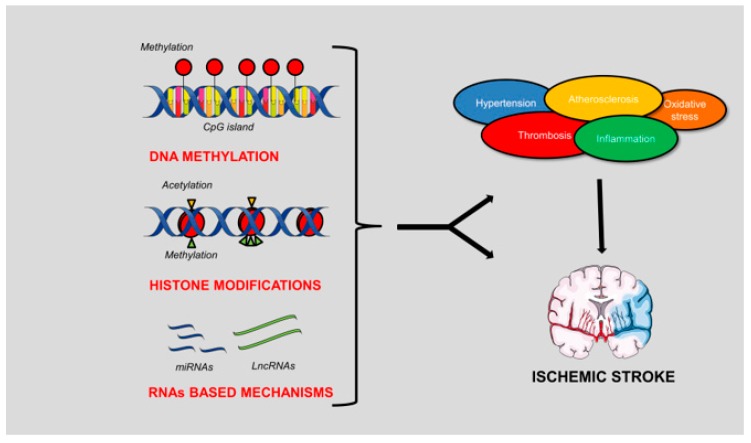
Schematic representation of the epigenetic mechanisms involved in the pathogenesis of ischemic stroke (IS). DNA methylation, histone modifications and RNA-based mechanisms can contribute either indirectly or directly to the development of the brain ischemic damage. In fact, both genes involved in the conditions predisposing to IS and genes directly involved in stroke pathogenesis are modulated by these mechanisms. The figure was made in part using tools provided by Servier Medical Arts.

In this review article, we will describe the different types of epigenetic mechanisms and their involvement in the pathogenesis of IS (Figure 1).

## 2. Overview of Epigenetic Mechanisms of Gene Regulation

Epigenetics includes the processes that modify gene expression without changing the DNA sequence [26,27]. The main epigenetic mechanisms include DNA methylation, histone modifications, and RNA-based mechanisms.

DNA methylation is a reversible modification which usually decreases gene transcription through a chemically stable modification carried out by DNA methyltransferase (DNMT) associated with the addition of a methyl group (-CH_3_) in the 5’ position of cytosine in the gene promoter. This modification prevents the interaction between transcription factors with their specific binding sites [28].

Histone modifications include acetylation and methylation of lysine residues in the terminal tail of histones H3 and H4. These changes are performed by histone acetyltransferases (HATs) and histone methyl transferases (HMTs), respectively [29,30]. These modifications modulate histone–DNA interactions by influencing the chromatin structure, thereby directing the accessibility of transcriptional regulators to DNA-binding elements. Histone acetylation, catalyzed by HATs, generally increases gene expression whereas histone deacetylation, through histone deacetylases (HDACs), inhibits gene expression. Histone methylation can either activate or inhibit the expression of genes and it usually involves the arginine residues [31].

Some interesting RNA-based mechanisms are those involving microRNAs (miRNAs) or long noncoding RNAs (LncRNAs). miRNAs are a group of small single-stranded noncoding RNAs of 18 to 22 nucleotides that have been identified in many organisms. miRNAs act as post-transcriptional regulators of gene expression. Once maturated, miRNAs interact with the 3’-untraslated region of a mRNA target (3’-UTR). Depending on the degree of complementarity between the miRNA and its target, gene silencing may occur either by mRNA degradation or by inhibition of translation. A single miRNA can regulate multiple gene targets [32]. miRNAs are involved in crucial biological processes including cell proliferation and differentiation, apoptosis, fat metabolism, and hematopoiesis [33,34]. They can be dysregulated in several pathological conditions including cardiovascular diseases [35].

In recent years, the interest in LncRNAs has also increased. LncRNAs are transcripts longer than 200 nucleotides, located within either the intergenic stretches or the overlapping antisense transcripts of protein coding genes. LncRNAs can regulate the expression of target genes by both transcriptional and post-transcriptional mechanisms [36]. It has been reported that LncRNAs are regulators of vascular function and their dysregulation contributes to cardiovascular diseases [37,38].

## 3. DNA Methylation in IS

The role of promoter DNA methylation in the pathogenesis of IS has been well investigated in recent years (Table 1) [39,40]. In general, the global level of DNA methylation is higher in animal models of IS and is associated with higher activity of DNMT in the brain. In fact, pharmacological inhibition of DNMT was shown to decrease DNA methylation, to reduce the infarct size and cerebral ischemic damage in the middle cerebral artery occlusion (MCAO) rat model [41,42,43].

In the same model, a decreased DNA methylation level of Na-K-Cl cotransporter 1 (*NKCC1*), a membrane protein involved in the active transport of sodium, potassium, and chloride, was associated with re-expression of brain NKCC1 mRNA and protein after neuronal injury [44]. Further studies are needed to define the specific role of NKCC1 in IS.

It is well established that disorders of the coagulation cascade predisposing to IS can cause either hemorrhagic or thrombotic lesions. For example, Hu et al. found that thrombospondin 1 (*THBS1*), an angiostatic factor also involved in platelet aggregation, was hypermethylated in an in vitro model of ischemia. This finding suggests that THBS1 may contribute to post-ischemic angiogenesis, a mechanism needed to repair brain damage following ischemia [45].

In humans, high levels of plasma homocysteine are known to be an independent risk factor for several vascular disorders including atherosclerosis, coronary artery disease, and stroke [46]. The metabolism of homocysteine is regulated by dietary factors, such as methionine and vitamins B, and by different enzymes, such as methylentetrahydrofolate reductase (MTHFR), cystathionine-beta-synthase (CBS) and methionine synthase MS (MS). CBS is the main enzyme involved in the conversion of homocysteine to cysteine. Dietary deficiencies and alterations in homocysteine metabolism are associated with higher stroke occurrence. Recently, Wang et al. described DNA hypermethylation in the *CBS* promoter and its association with increased risk of stroke in human populations. Hypermethylation of *CBS* leads to low enzyme activity, plasma homocysteine accumulation, and increased susceptibility to stroke [47]. High levels of plasma homocysteine are also associated with DNA hypermethylation of thrombomodulin (*TM*) in patients with IS. TM is an integral membrane protein expressed on the surface of endothelial cells. It possesses anticoagulant properties by acting as a thrombin receptor. The soluble form of TM plays an important role in the regulation of intravascular coagulation. For this reason, TM exerts a protective effect against cerebral ischemia. In fact, DNA hypermethylation of *TM* promoter was found to correlate with high level of homocysteine, with increased endothelial damage and increased risk of IS [48].

Epidemiological studies have shown the role of dyslipidemia as an important risk factor for IS [49]. In this regard, elevated levels of both triglycerides and low-density lipoprotein (LDL)-cholesterol were associated with an increased risk of stroke, whereas high-density lipoprotein (HDL) cholesterol levels showed an inverse correlation [50,51,52,53]. Several studies suggest the role of apolipoprotein E (ApoE) in IS. ApoE is a plasma lipoprotein involved in lipid metabolism that interacts with the LDL receptor [54]. Polymorphisms of the ApoE gene (*APOE-ε*) were shown to be frequent in subjects with atherosclerosis [54], Alzheimer’s disease [55], and IS [56]. A recent study also demonstrated the association between the methylation status of the *ApoE* gene promoter and atherosclerotic cerebral infarction (ACI), a common form of stroke. Specifically, DNA hypermethylation of *ApoE* promoter repressed the expression of the gene. The latter correlated with lower levels of both HDL cholesterol and folate, and with higher levels of homocysteine. The hypermethylation of *ApoE* can be prevented by reducing homocysteine level with folate administration [57].

Higher level of DNA methylation of cyclin dependent kinase inhibitor 2B (*CDKN2B*), a gene involved in the pathogenesis of both atherosclerosis and arterial calcification, was associated with increased susceptibility to calcification of the arteries in patients with IS [58].

Finally, the expression of inflammatory mediators is also influenced by epigenetic mechanisms. Vascular cell adhesion protein 1 level (VCAM-1) represents a marker of cardiovascular diseases since it is rapidly expressed in pro-atherosclerotic conditions [59,60]. Baccarelli et al. showed that the hypomethylation of long interspersed nucleotide elements (LINE-1) was associated with increased levels of VCAM-1 in elderly individuals. In the same study, the authors reported that the association between LINE-1 hypomethylation and VCAM-1 expression was significant only in subjects without prevalent ischemic heart disease or stroke. Instead, no correlation was found in patients with heart disease and stroke. The latter evidence suggests that the association between LINE hypomethylation and VCAM-1 expression may be considered as an early event in the etiology of cardiovascular and cerebrovascular diseases [61]. The hypomethylation of TNF receptor-associated factor 3 (*TRAF3*) and of protein phosphatase 1A (*PPM1A*) is also related to increased stroke occurrence in patients treated with antiplatelet drugs [62,63].

## 4. Histone Modifications in IS

Studies in both humans and animal models highlighted the role of histone modifications in chromatin remodeling and their involvement in various brain disorders including IS [64]. Several groups identified a general decrease of histone H3 [65] and H4 [66] acetylation level in animal models of stroke, which was associated with severe brain damage. Since epigenetic changes can be reversed, the administration of HDAC inhibitors was reported to be a valid approach to decrease brain injury. In the MCAO models, the administration of valproic acid or sodium butyrate, two widely used HDAC inhibitors, was found to either preserve or restore the normal acetylation level of H3 and H4 histones. These effects contributed to a substantial reduction of infarct volume and to the improvement of brain damage. Furthermore, a significant increase of Hsp70, a prosurvival heat shock protein, and a parallel decrease of proapoptotic protein caspase-3 were reported in MCAO models treated with HDAC inhibitors [67]. Recently, a relationship between HDAC4 and IS was highlighted. Kong et al. described that HDAC4 was reduced in neuronal cells isolated from MCAO rat model and subjected to oxygen-glucose deprivation (OGD) [68]. Low levels of HDAC4 were associated with alterations in cell proliferation and angiogenesis [69] and with loss of integrity of the blood–brain barrier (BBB) [70]. Exogenous administration of HDAC inhibitors rescued H4 acetylation level, restored BBB integrity, and reduced infarct volume in the MCAO rat [71].

HDAC inhibition exerts a protective effect also against inflammatory reactions in IS [72]. Indeed, in primary microglial cells isolated from the MCAO mouse model, HDAC inhibition with sodium butyrate was reported to increase the acetylation of lysine 9 within H3 histone (H3K9ac), along with the downregulation of different proinflammatory mediators, such as interleukin 6 (IL-6), inducible nitric oxide synthase (iNOS), tumor necrosis factor-α (TNF-α), and with a parallel upregulation of the anti-inflammatory mediator interleukin 10 (IL-10) [40].

Despite promising results obtained in preclinical studies, the use of HDAC inhibitors have not yet provided satisfactory results in patients with IS. First of all, most specific inhibitors of HDAC are not permeable to the BBB; secondly, unspecific HDAC inhibitors were associated with several adverse effects such as weight loss, taste disturbances, electrolyte changes, and cardiac arrhythmias in stroke Phase I/II clinical studies [73]. However, Brookes et al. reported the protective effects of sodium valproate, a nonspecific inhibitor of HDAC9, in patients with previous stroke or transient ischemic attack. They found that the administration of sodium valproate strongly reduced the risk of recurrent stroke [74].

## 5. RNA-Based Mechanisms in IS

Human and animal studies revealed alterations of miRNA levels in several pathological conditions, such as hypertension, dyslipidemia, atherosclerosis, and inflammation, that predispose to IS [75]. In addition, modifications of miRNA levels were identified in different stages of stroke suggesting their potential use for diagnostic, prognostic, and therapeutic purposes [76,77,78].

Studies performed in the MCAO rat model found a significant increase of both cerebral and plasma levels of miR-124, reaching a peak at 24 h after stroke development, compared to the control group. These results suggest that the plasma level of miR-124 can be used to monitor brain ischemic damage [79]. In the same rat model, Dharap et al. studied the brain profile of 238 miRNAs and found several up and downregulated miRNAs at different times of reperfusion. A bioinformatic analysis revealed that the deregulated miRNAs target the mRNAs of several genes involved in IS. Among them, there are genes belonging to the inflammatory pathway [interleukin 1β (*IL1β*) and *IL6*, intercellular adhesion molecule 1 (*ICAM1*), cyclooxygenase 2 (*COX2*)], to neuroprotection [heat shock protein 27 (*HSP27*), peripheral-type benzodiazepine receptor (*PTBR*), manganese superoxide dismutase (*Mn-SOD*), insulin-like growth factor binding protein 3 (*IGF-BP3*)], to transcription regulation [nuclear factor kappa-light-chain enhancer of activated B cells (*NF-kB*), hypoxia inducible factor 1 (*HIF1*), early growth inhibitor 1 (*Egr1*), activating transcription factor 3 (*ATF3*)] [80]. These processes collectively play a fundamental role during post-ischemic brain remodeling.

In the stroke prone spontaneously hypertensive rat (SHRSP), a suitable rat model of human stroke [81], a reduced miR-122 level behaves as a marker of stroke and is associated with reduced endothelial integrity, increased apoptosis and inflammation [82]. In the same rat model, the brain expression of uncoupling protein 2 (UCP2) is regulated by miR-503. UCP2 is a mitochondrial anion carrier protein that exerts an antioxidant effect in several tissues [83]. High-salt diet fed SHRSP showed a significant upregulation of brain miR-503 level compared to the control strain, the stroke resistant spontaneously hypertensive rat (SHRSR), with a consequent decrease of brain UCP2 expression and increased stroke occurrence [17].

Recently, the association between microRNA-21 and stroke was also investigated. miR-21 is upregulated in several diseases such as diabetes [84], hypertension [85], other cardiovascular diseases, and cancer [86]. Several reports suggest that mir-21 regulates cell proliferation, apoptosis, invasion, and migration by targeting genes including phosphate and tensin homolog (*PTEN*), reversion inducing cysteine rich protein with kazal motifs (*RECK*), B-cell lymphoma 2 (*Bcl-2*), and programmed cell death 4 (*PDCA4*) [87]. The level of miR-21 was found to be increased in the MCAO rat model and in stroke patients. In the MCAO model, an increase of miR-21 level was related to the downregulation of Fas Ligand (Faslg)*,* a member of the tumor necrosis factor super-family and a predicted target gene of miR-21. Faslg can trigger apoptosis by binding to Fas cell surface death receptor (FAS). Faslg is an important cell death regulator and its downregulation exerts a protective effect in neuronal cells [88]. Plasma levels of miR-21 were also found to be significantly upregulated in patients with IS but not in patients with transient ischemic attack. It is likely that miR-21 may represent a discriminative biomarker for stroke subtypes [89].

Studies performed in young stroke patients also demonstrated a specific profile of miRNAs expression. The deregulated miRNAs included mostly those regulating angiogenesis, neuronal, and vascular functions [90].

Recently, miR-335 has also emerged as an important biomarker in IS. A low plasma level of miR-335 was found in patients with acute IS and it correlated with the increase of plasma calmodulin (CaM), a direct target of miR-335 [91]. Calmodulin is a protein involved in many calcium-mediated processes and plays an important role in the mechanisms of ischemic brain injury and of stroke progression [92]. These results support the miR-335 as a useful noninvasive circulating biomarker of stroke.

Chen et al. studied the relationship between miR-211 and angiopoietin1 (ANG-PT1) in a large cohort of IS patients [93]. ANG-PT1 is a protein involved in endothelial cell survival and angiogenesis [94,95] with a binding site for mir-211 localized at 3’-UTR. This binding site matches with a gene polymorphism (rs2507800 A > T). Carriers of the A allele (wild-type) bind normally the miR-211 and show a downregulation of ANG-PT1 level. On the other hand, the presence of the mutant T allele reduces the miR-211 binding with a significant increase of ANG-PT1 level, which in turn contributes to vascular damage and increased stroke susceptibility [93].

Our group demonstrated that the T2238C variant of the atrial natriuretic peptide (ANP) gene, a nonmodifiable risk factor for cardiovascular diseases including stroke, modulates ApoE level through miR-199 in vitro. The miR-199 up-regulation induced by C2238/αANP, and not by the wild type ANP, produced a downregulation of ApoE associated with decreased cell viability and increased apoptosis, necrosis, and inflammation in human vascular smooth muscle cells [96]. These results support the growing importance of miRNAs in the pathogenesis of stroke.

In recent years, the interest on LncRNAs has also increased. LncRNAs act as important regulatory factors in IS by modulating cell survival, inflammation, and angiogenesis. New technologies allowed the discovery of more than 200 LncRNAs differentially expressed in the brain of animal models and in the blood of IS patients [97,98]. For example, the role of LncRNAs ANRIL (antisense non-coding RNA in the INK4 Locus), MALAT1 (metastasis associated lung adenocarcinoma transcript 1), MEG3 (maternally expressed 3) and TUG1 (taurine up-regulated 1) was investigated in preclinical models of IS [99,100]. Expression of ANRIL was found significantly increased in the brain of rat models of stroke where it promoted angiogenesis through the activation of vascular endothelial growth factor (VEGF) and of its receptor ferritin light chain-1 (FLT-1). ANRIL was found to regulate also inflammation through NF-kB signaling [101].

MALAT1, MEG3, and TUG1 are LncRNAs involved in the regulation of cell proliferation, inflammation, and apoptosis. Abnormalities of the regulation of these processes are involved in the pathogenesis of IS through the causation of neuronal injury. Recently, MALAT1 was found to be significantly increased in the brain of the MCAO mouse model as an adaptive response following the ischemic insult. Consistently, MALAT1 knockout mice undergoing MCAO showed an exacerbated brain injury, along with the increase of proapoptotic and inflammatory factors. The latter evidence suggests that MALAT1 plays a protective role in IS [102].

The LcnRNA MEG3 was shown to be overexpressed in the brain of the MCAO mice, along with the upregulation of p53 and of 12/15-Lipoxygenase (12/15-LOX), two activators of cell death [103,104]. Specifically, MEG3 was found to interact with p53 during ischemia, allowing the activation of neuronal cell apoptosis. The inhibition of MEG3 interaction with p53 suppressed neuronal apoptosis and reduced infarction volume [103]. In a separate study, MEG3 upregulation was associated with the increase of 12/15-LOX and with increased neuronal cell death [105]. Overall, these findings suggest that MEG3 exerts deleterious effects during brain ischemia.

The LncRNA TUG1 also regulates apoptosis in different cell types [106,107]. Regarding its role in IS, Chen et al. reported TUG1 overexpression in the brain of the MCAO rat model. In the same study, TUG1 was found to bind miR-9 in primary neurons subjected to oxygen-glucose deprivation (OGD), with the consequent decrease of its activity. miR-9 acts as an anti-apoptotic factor by targeting the pro-apoptotic protein Bim-1. Inhibition of TUG1 rescued neuronal survival during OGD by boosting miR-9 activity [108].

In humans, different studies have characterized the profile of approximately 3000 LncRNAs by using several technologies. The expression level of hundreds of LncRNAs was altered in peripheral blood of patients with IS. These results suggest that lncRNAs may have a potential role in stroke development and progression [109].

## 6. Conclusions

Several studies performed in animal models of stroke and in human patients contributed to elucidate the complex genetic and molecular mechanisms involved in the pathogenesis of IS. The epigenetic mechanisms are emerging as novel, fundamental factors involved in the regulation of the expression of stroke-related genes. Epigenetic modifications, differently from change in gene sequence, are reversible. Pharmacological inhibitors of DNA methyltransferases, histone acetyltransferases, and histone deacetylases, the main enzymes involved in epigenetic modifications, can be used to change the expression level of a gene of interest. Interestingly, some of these inhibitors are already used in animal models of stroke [110] and they are available in clinical practice for the treatment of hematological diseases [111]. The antagomirs administration induces a neuroprotective effect in animal models of stroke [112]. This approach appears to be promising in order to inhibit miRNAs for therapeutic purposes [113].

Although the development of molecules regulating the epigenetic processes may result difficult and expensive, this new approach could reveal very useful for the development of novel pharmacological therapies against IS in the future.

## Figures and Tables

**Table 1:** DNA methylation in IS.

Study Model	Condition/Effects	Outcomes	References
MCAO rat	Pharmacological inhibition of DNMT	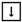 Infarct size 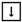 Cerebral ischemic damage	[41,42,43]
MCAO rat	Increased demethylation of *NKCC1*	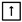 Cerebral ischemic injury	[44]
Rat endothelial cells under oxygen-glucose deprivation (OGD)	Hypermethylation of *THBS1*	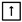 Angiogenesis	[45]
Hypertensive and stroke patients	Hypermethylation of *CBS*	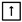 Homocysteinemia 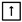 stroke occurrence	[47]
Stroke patients	Hypermethylation of *TM*	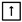 Vascular endothelial damage 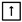 Risk of IS	[48]
Meta-analysis study of stroke patients Stroke patients	Hypermethylation of *ApoE*	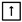 Lipoprotein accumulation 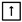 Atherosclerotic cerebral infarction	[56,57]
Stroke patients	Hypermethylation of *CDKN2B*	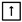 Aortic arch calcification	[58]
Elderly patients	Hypomethylation of LINE-1	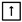 VCAM-1 level	[61]
Stroke patients receiving antiplatelet drugs	Hypomethylation of *TRAF3* and *PPM1A*	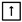 Stroke occurrence	[62,63]

Legend: 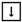 indicates decrease whereas 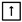 indicates increase.
